# Moderate exercise protects against joint disease in a murine model of osteoarthritis

**DOI:** 10.3389/fphys.2022.1065278

**Published:** 2022-12-05

**Authors:** C. Huesa, L. Dunning, K. MacDougall, M. Fegen, A. Ortiz, K. McCulloch, S. McGrath, G. J. Litherland, A. Crilly, R. J. Van ‘T Hof, W. R. Ferrell, C. S. Goodyear, J. C. Lockhart

**Affiliations:** ^1^ School of Infection and Immunity, University of Glasgow, Glasgow, United Kingdom; ^2^ School of Health and Life Sciences, University of the West of Scotland, Paisley, United Kingdom; ^3^ Institute of Life Course and Medical Sciences, University of Liverpool, Liverpool, United Kingdom

**Keywords:** exercise, osteoarthritis, bone, cartilage, destabilisation of medial meniscus model, subchondral bone

## Abstract

Exercise is recommended as a non-pharmacological therapy for osteoarthritis (OA). Various exercise regimes, with differing intensities and duration, have been used in a range of OA rodent models. These studies show gentle or moderate exercise reduces the severity of OA parameters while high intensity load bearing exercise is detrimental. However, these studies were largely conducted in rats or in mouse models induced by severe injury, age or obesity, whilst destabilization of the medial meniscus (DMM) in mice has become a widely accepted model due to its lower variability, moderate progression and timescale. The present study was undertaken to provide insight into the effect of moderate exercise on early joint pathology in the DMM mouse model. Exercise was induced a week after induction by forced wheel walking for three or 7 weeks. Joints were analyzed by microcomputed tomography and histology. Assessment of skeletal parameters revealed that exercise offered protection against cartilage damage after 7 weeks of exercise, and a temporary protection against osteosclerosis was displayed after 3 weeks of exercise. Furthermore, exercise modified the metaphyseal trabecular microarchitecture of the osteoarthritic leg in both time points examined. Collectively, our findings corroborate previous studies showing that exercise has an important effect on bone in OA, which subsequently, at 8 weeks post-induction, translates into less cartilage damage. Thus, providing an exercise protocol in a surgical mouse model of OA, which can be used in the future to further dissect the mechanisms by which moderate exercise ameliorates OA.

## Introduction

Osteoarthritis (OA) affects ∼80% of people aged over 50. It is characterized by structural and functional changes in articular joints, with concomitant pain and loss of joint mobility that significantly impairs quality-of-life. To delay rapid progression of OA, international guidelines recommend therapeutic exercise (Fernandes et al., 2013; McAlindon et al., 2014; Bannuru et al., 2019). Numerous studies have shown that exercise regimes, especially aerobic and strengthening, when monitored closely and performed regularly, lead to an improvement in joint movement, physical activity and pain (Fransen et al., 2015; Barton et al., 2021; Raposo et al., 2021). Exercise also induces weight loss, reduces inflammation (Messier et al., 2013; Onu et al., 2021) and has an important positive psychological impact in humans (Hurley et al., 2018; Wang and Ashokan, 2021). Whilst clinical studies consistently support exercise as a possible treatment for OA, there is a lack of understanding of how exactly exercise improves the joint.

To better understand the effects of exercise in the osteoarthritic joint, the last decade has seen an increase in studies of exercise on rodent OA *in vivo* models. The exercise regimes and the models of OA induction are varied (Table 1). In male rats, Iijima and others surgically induced OA *via* the destabilization of the medial meniscus (DMM) (Glasson et al., 2007), which in rodents results in progressive development of OA with cartilage damage, osteosclerosis, variable synovitis, ligament damage/calcification and osteophyte formation (Glasson et al., 2007; Jackson et al., 2014; Huesa et al., 2016). Studies utilizing DMM induction of OA on rats followed by treadmill exercise showed that 1) gentle treadmill walking prevented OA changes specially subchondral bone growth (Iijima et al., 2015), 2) longer rest before starting exercise was more beneficial (Iijima et al., 2016) and finally 3) that intense treadmill running is more detrimental to the joint (Iijima et al., 2017). Forced mobilization on a rotating cylinder in a rat transection of the anterior cruciate ligament (ACL-T) model induced increased cartilage degradation, subchondral plate failure and earlier subchondral bone sclerosis, suggesting that repetitive load-bearing exercise is detrimental (Appleton et al., 2007). However, a more severe model of OA on rats (ACL-T and DMM together) showed moderate to reduce progression of OA and this improvement was enhanced by reducing body weight load to 60%. In mice, exercise has also been explored, where OA was induced by high fat diet (Griffin et al., 2012; Hahn et al., 2021), age (Lapveteläinen et al., 1995), ACL rupture (Hsia et al., 2021), spaceflight/limb unloading (Kwok et al., 2021), ACL-T (Oka et al., 2021) or DMM exacerbated by restricted movement (Kim et al., 2013). Similar to the rat model, high intensity exercise resulted in aggravated OA whilst moderate treadmill or voluntary wheel exercise improved OA parameters in the joint.

**TABLE 1:** Published exercise and load studies on rodent models of osteoarthritis, summarizing the experimental set up and intensity of exercise, loading or unloading protocols and the effects these had in the joint.

Authors	Year of publication	Model	OA induction	Type of exercise	Speed	Duration	Result
Appleton et al.	2007	Male Rat	ACL-T and partial meniscectomy	Rotating cylinder	4 rpm	30 min 3 days/wk up to 20 weeks	Forced mobility accelerates OA onset and severity
Hao et al.	2021	Male rat	ACL-T + DMM	Treadmill running vs. Treadmill running with 60% body weight	15 m/min	30 min/d, 5 days/wk for 4 weeks	Exercise reduced progression of OA and this improvement was enhanced by reducing body weight load to 60%
Iijima et al.	2015	Male rat	DMM	Gentle treadmill walking	6 m/min	From day 2 for 1, 2 and 4 weeks 30 min 5 days/wk	Showed prevention of OA changes, especially in the subchondral bone
Iijima et al.	2016	Male rat	DMM	Moderate treadmill walking	12 m/min	from day 2 for 4 weeks, from week 4 through 8 weeks, or from day 2 through 8 weeks 30 min 5 days/wk	Longer rest from induction showed better prevention from OA changes
Iijima et al.	2017	Male rat	DMM	Moderate vs. high speed treadmill	12 or 24 m/min	From 4 weeks to 56 weeks 30 min 5 days/wk	Intense treadmill exercise is detrimental to the OA joint
Griffin et al.	2012	Male mice	High fat diet	Voluntary running wheel	N/A	from 20 to 24 weeks of age	Exercise reduced progression of OA.
Hahn et al.	2021	Male mice	High fat diet	Voluntary running wheel	N/A	from week 26–52	No improvement to articular cartilage and synovial fluid metabolite links to subchondral bone structure
Hsia et al.	2021	Female mice	Non-invasive ACL rupture	Hind limb unloading		One week after induction then resume normal activity up to 4 weeks	Less osteophyte formation in unloaded group
Kim et al.	2013	Male and female mice	DMM + restricted cage	Treadmill running	15 m/min	6 days/wk	Increased severity of OA lesions in exercise group
Kwok et al.	2021	Male mice	space flight or limb unloading	35 days of space flight or hind limb unloading followed by exercise 1) climbing or 2) treadmill	10.2 m/min	3 days/wk up to day 80 after unloading or space flight	Either spaceflight or unloading generated cartilage and menisci degradation. Exercise reduced cartilage degradation and improved thickness
Lapvetelainen et al.	1995	Male mice	Not induced	Treadmill running	13.3 m/min	From 2 months old up to 6, 10, 14 and 18 months old. 75 min 5 days/wk	Running increased the incidence and severity of OA.
Oka et al.	2021	Male mice	ACL-T	High speed treadmill	18 m/min	30 min 3 days/wk for 4 weeks	Deterioration of the cartilage due to exercise

One commonality of these mouse models is that the induction of OA is either very long or more severe than the standard, more commonly used, DMM. DMM on mice offers a widely accepted model due to its moderate progression, reproducibility, and timescale, better reflecting the course of a large proportion of human post-traumatic OA cases, as well as offering the availability of transgenics. In this study, we investigated the effects of moderate forced exercise in the mouse DMM model. To do so, we generated an exercise protocol that allows for a period of recovery after injury/induction before a type of exercise that mimics long daily walks. We then assessed joint osteoarthritic structural changes, such as cartilage damage, inflammation, and bone micro-structure. In this study we sought to establish and characterize an exercise model of OA in mice that would facilitate investigation of the mechanisms underpinning the amelioration of OA by moderate exercise.

## Methods

### Animals, induction of OA and exercise

DMM (Glasson et al., 2007) was performed on 10-week-old male C57BL/6 mice weighing on average 26.0 ± 1.4 g. A total of 42 mice were purchased (Envigo, United Kingdom) and placed in plastic cages with sawdust bedding (4–five animals per cage) in a 12-h light/dark cycle at constant temperature. Animals were monitored daily, allowed to move freely in cages and provided free access to food, water and environmental enrichment. A week before surgery, all mice were tested for a few minutes on regulated rotating wheels (Campden Instruments Ltd. Loughborough) and those capable of using the wheels were selected for the exercise group. Exercise was set for 850m/day at a speed of 3.8 m/min, with 18s break every 4min. The total distance was divided in two sessions with a 2–3 h break in between. At surgery animals were given analgesics (Buprenorphine, 0.1 mg/kg). Exercise commenced 1-week post-surgery and continued 5 days/week for three or 7 weeks. Experimental groups are indicated in Supplementary Table S1. At endpoint, blood and tissues were collected. Legs were harvested for assessment *via* microcomputed tomography (μCT) and histology. Subcutaneous, gonadal, and brown fat pads, together with quadriceps and soleus muscles, were dissected and weighed (wet weight). The analysis was conducted blind; groups were only revealed at the end of all analysis. All procedures were in accordance with Home Office regulations and experimental design was pre-approved by the Ethical Review Committee at the University of Glasgow. The study is reported in accordance with ARRIVE guidelines (https://arriveguidelines.org).

### Microcomputed tomography

Knees were fixed (4% paraformaldehyde) for 24 h and stored (70% ethanol). Joints were analyzed by μCT using the Skyscan 1272 (Bruker, Belgium; 0.5 aluminium filter, 50kV, 200µA, voxel size 4.57 μm, 0.5° rotation angle). Scans were reconstructed in NRecon software (Bruker, Belgium), with stacks analyzed: 1) osteophytes identified in three-dimensional reconstructions and volume measure by selecting a region of interest (ROI) in 2D stacks as previously described (Huesa et al., 2016) and 2) subchondral bone within the tibial epiphysis was selected (from the growth plate to subchondral plate) in a volume of interest (VOI) under the increased loading area (Das Neves Borges et al., 2014). 3) Tibial metaphyseal trabecular bone was analyzed in a stack of 200 slices taken ∼230 µm from the lower end of the growth plate.

### Histology and scoring

After µCT, joints were decalcified (Formical 2000; Decal Chemical, United States) overnight, embedded in paraffin wax and coronal sections (5 μm) cut, and stained with haematoxylin and Safranin-O/Fast-Green. Using a validated scoring system (Glasson et al., 2010) ranging from 0 (normal) to 6 (>80% loss of cartilage), the tibial quadrant in 8–10 sections from each mouse was graded by two scorers blinded to the specimens, with scores averaged. There was good agreement between scorers; intraclass correlation coefficient of 0.9 (95% CI 0.82–0.95), mean difference in score being 0.12 (95% CI 0.19–0.33). Synovitis was assessed using a validated scoring system (Jackson et al., 2014). This was modified to focus on pannus formation, synovial membrane thickening and sub-synovial hyperplasia. There was agreement between scorers; intraclass correlation coefficient of 0.88 (95% CI 0.79–0.94), mean difference in score of 0.002 (95% CI −0.07 to 0.35).

### Nocturnal activity

Nocturnal activity was measured by placing a mouse in an activity cage (Activmeter, Bioseb, France). Activity monitoring was conducted in the last 2 weeks of the 8-week protocol. Cage activity measurements represent averaged total movements throughout 16 h of recording.

### Statistics

Data were tested for normality with a Shapiro-Wilk test (GraphPad Prism, v9.4.1) and presented as mean ± standard deviation or showing each data point highlighting the mean/median. Differences were statistically analyzed by *t*-test or two-way analysis of variance (ANOVA) with Bonferroni correction for multiple comparisons. Non-normal distribution or datasets too small to test for normality were compared by non-parametric tests such as Mann-Whitney test for un-paired data and Wilcoxson for paired data. Data is available upon request.

## Results

### Moderate exercise shows signs of physiological changes

**FIGURE 1:**
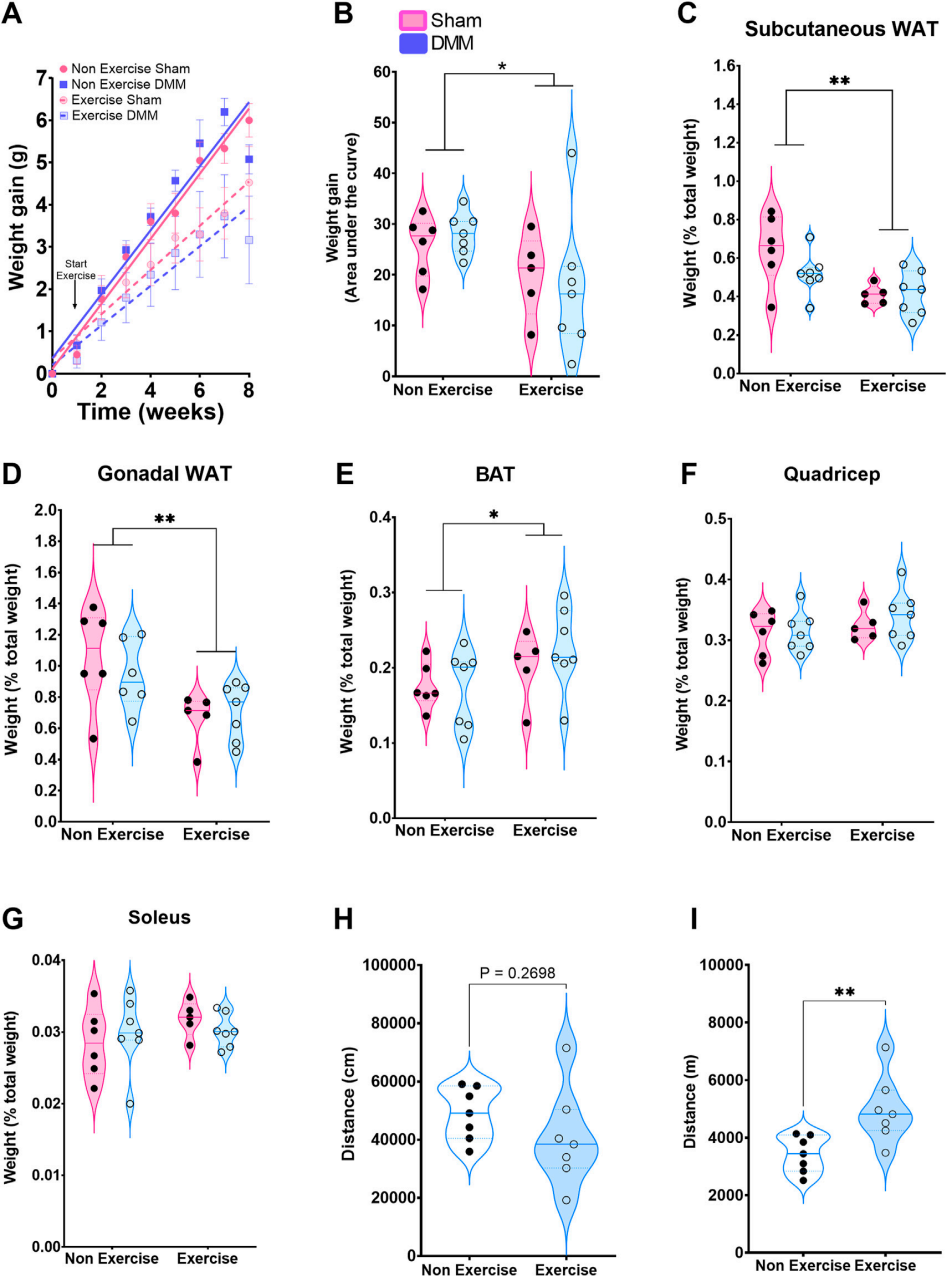
Overall effect of the exercise protocol on weight expressed as weight gain mean ± SEM **(A)** and area under the curve **(B)**. Subcutaneous **(C)**, gonadal **(D)** and brown **(E)** adipose tissue weight expressed as a percentage of total weight. Quadricep **(F)** and soleus **(G)** muscle weight as a percentage of total body weight. **(H)** Overnight distance travelled in DMM mice. **(I)** Calculated weekly distance based on overnight plus forced exercised distance. Arrow in **(A)** indicates start of exercise protocol. Weight gain was analyzed by mixed-effects model with time, surgery and exercise as factors. Time and exercise were significant (p < 0.0001). AuC, fat pad and muscle weight were analyzed with a 2-way ANOVA with Bonferroni correction. Movement in DMM groups was analyzed by Standard student t-test. *p < 0.05, **p < 0.01.

Exercise induced a reduction in weight gain regardless of the type of surgery (DMM/Sham, two-way ANOVA, *p* = 0.006 Exercise vs. Non exercise), which was evident 5 and 6 weeks after initiation of exercise (Figure 1A&B). This was reflected in the loss of white adipose tissue (WAT), measured as percentage tissue weight to total body weight (Figure 1C&D). Subcutaneous and gonadal WAT were significantly lower in the exercise group 8-week post-DMM surgery, whilst inter-scapular brown adipose tissue (iBAT, Figure 1E) was significantly higher in the exercise group, regardless of surgical intervention. No changes in muscle mass were noted (Figure 1F&G). Recognizing that forced exercise might induce changes in the overall activity, we measured overnight activity in the DMM group comparing exercised to non-exercised (*n* = 6 per group). There was no significant difference in nocturnal distance travelled between the groups, and therefore forced exercise did not have a meaningful impact on the total amount of voluntary exercise/activity undertaken (Figure 1H). To calculate the weekly distance travelled we added 7 times the voluntary distance travelled to 5 times the calculated distance of the forced exercise (Figure 1I). This resulted in an increased mean weekly distance travelled (1.5 times higher) within the exercise group. Thus, exercise increased physical activity by 50%. We did not observe significant changes in pain behaviors as measured by dynamic weight bearing (Supplementary Figure S1).

### Exercise reduces subchondral bone osteosclerosis at 4 weeks

**FIGURE 2:**
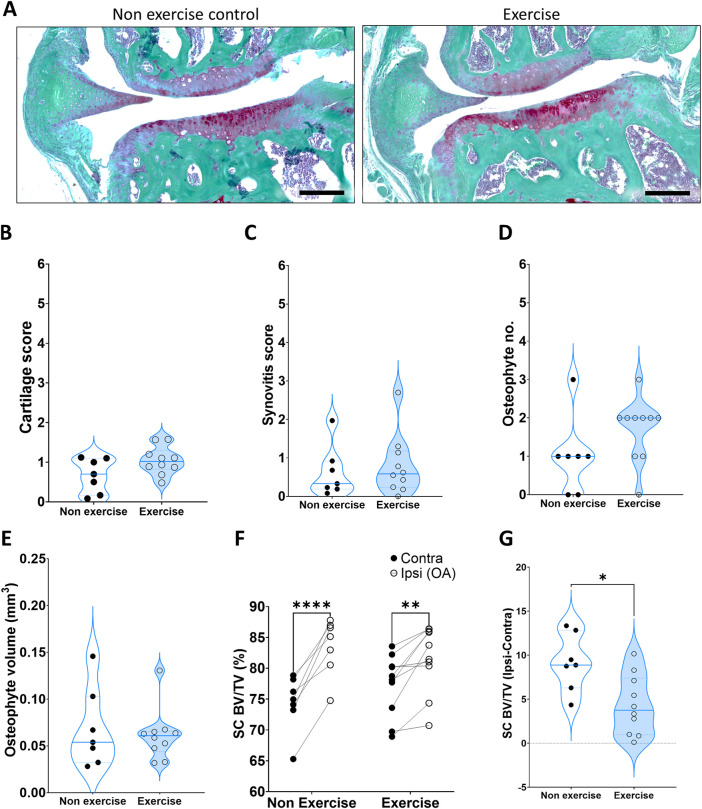
Disease parameters on mice 4 weeks after induction of OA. **(A)** Representative images of the DMM joint at 4 weeks, stained with SafraninO for cartilage and Fast Green for bone. **(B)** Cartilage score. **(C)** Synovitis score. **(D)** Osteophyte number. **(E)** Osteophyte volume. **(F)** Comparison of medial subchondral bone compartment density (% BV/TV) between the operated ipsilateral (Ipsi) and control contralateral (Contra) legs. **(G)** Change in tibial subchondral bone sclerosis (Ipsi–Contra). Comparison between exercise and non-exercise groups was done with a t-test unless data was not normally distributed, in which case it was then compared by a Mann-Whitney test. Paired comparisons were conducted via a paired t-test. *p < 0.05, **p < 0.01, ***p < 0.001.

Moderate exercise did not lead to any significant histological changes in articular cartilage damage (Figures 2A,B) or synovitis (Figure 2C) at the early 4-week time point. The number of osteophytes, measured as protruding bone formation on the medial side of the subchondral bone, was also not statistically significant at 4-week (Figure 2D). Despite this, 70% of exercise samples had two or more osteophytes whilst only one sample out of 7 (14%) in the non-exercise group had two or more osteophytes. A Fisher exact test where data was separated into two groups, 1) one osteophyte or less and 2) two osteophytes or more, indicated the exercise group was significantly different from the non-exercise group (*p* = 0.0498). This indicates increased osteophyte formation during the initial phase of the model, when the subchondral bone is adapting to the new loading resulting from the destabilization. This increase in osteophyte formation may be indicative of faster subchondral bone expansion, yet we found no changes in subchondral osteophyte volume at this time point (Figure 2E). Subchondral osteosclerosis, measured as the ratio-metric comparison of subchondral bone % BV/TV in the medial tibial compartment of the knee and the contralateral leg (SC % BV/TV, Figure 2F and Table 2), was evident in all DMM groups. Yet, osteosclerosis was significantly reduced in exercised mice (Table 2; Figure 2G). DMM also induced changes to metaphyseal trabecular bone, but only in the exercise group where the ipsilateral leg had less trabecular bone, due to a decrease in the number of trabeculae, which were also more plate-like (structural model index, SMI, Table 2).

**TABLE 2:** MicroCT analysis of trabecular and subchondral bone changes in the DMM groups. BV/TV = Bone volume/Tissue volume. Tb.Th = trabecular thickness. Tb.No = Trabecular number. Tb.Sp. = Trabecular space. Tb.Pf = Trabecular pattern factor (connectivity). SMI = Structural model index (shape). DA = Degree of anisotropy (organization). SC = Subchondral. Bone scl = bone osteosclerosis. Each time point was analyzed with a Two-Way ANOVA, comparing relative changes to the contralateral leg and also interrogating the effect of exercise. Data was also compared between exercise and non-exercise within the DMM joint normalized against the contralateral leg (NE vs. E). *p* values under 0.05 were considered significant.

4 weeks	Non exercise							Exercise							p
	Contralateral			Ipsilateral			p	Contralateral			Ipsilateral			p	Ne vs. E
BV/TV (%)	12.3	±	0.84	13.2	±	0.71	0.155	13.2	±	0.71	12.9	±	0.77	0.045	0.937
Tb.Th (µm)	12.0	±	0.38	12.1	±	0.3	0.661	12.1	±	0.3	11.9	±	0.35	0.940	0.644
Tb.Sp (µm)	55.4	±	3.39	52.9	±	2.47	0.226	52.9	±	2.47	53.9	±	1.93	0.451	0.437
Tb.N (µm−1)	0.010	±	0.00051	0.011	±	0.00054	0.076	0.010	±	5 × 10-4	0.011	±	0.00045	0.026	0.894
Tb.Pf (µm−1)	0.097	±	0.00745	0.093	±	0.0049	0.392	0.093	±	0.005	0.093	±	0.0071	0.055	0.542
SMI	1.97	±	0.0663	1.94	±	0.0548	0.568	1.94	±	0.055	1.88	±	0.0584	0.008	0.113
DA	2.60	±	0.0937	2.76	±	0.778	0.292	2.76	±	0.778	2.65	±	0.0494	0.093	0.961
SC bone scl (%)	74.4	±	1.71	83.5	±	1.74	3 × 10-4	77.3	±	1.58	81.6	±	1.70	0.003	0.010
SC Tb.Th (µm)	28.0	±	1.23	33.1	±	1.14	0.003	28.7	±	1.24	34.5	±	1.58	5 × 10-6	0.536
															

8 weeks	Non exercise							Exercise							p
	Contralateral			Ipsilateral			p	Contralateral			Ipsilateral			p	NE vs. E
BV/TV (%)	18.0	±	0.838	18.1	±	0.724	0.820	15.3	±	1.36	15.69	±	1.45	0.338	0.786
Tb.Th (µm)	56.7	±	1.434	56.5	±	1.587	0.804	54.7	±	1.75	55.21	±	2.16	0.632	0.582
Tb.Sp (µm)	199.2	±	7.969	196.0	±	4.177	0.582	209	±	7.86	215	±	7.54	0.219	0.226
Tb.N (µm−1)	0.0032	±	0.00016	0.0032	±	0.00012	0.780	0.0028	±	2 × 10-4	0.0028	±	0.00021	0.362	0.946
Tb.Pf (µm−1)	0.0164	±	0.00073	0.0158	±	0.00067	0.480	0.0201	±	0.002	0.019	±	0.002	0.039	0.409
SMI	1.70	±	0.06386	1.64	±	0.047	0.328	1.8903	±	0.111	1.79	±	0.12	0.017	0.635
DA	2.22	±	0.05572	2.30	±	0.039	0.359	2.17	±	0.094	2.30	±	0.10	0.017	0.552
SC bone scl (%)	84.3	±	1.16	91.7	±	1.04	2 × 10-2	78.6	±	2.97	85.7	±	2.84	0.001	0.617
SC Tb.Th (µm)	30.2	±	1.05	38.4	±	1.24	2 × 10-5	28.6	±	1.65	33.84	±	1.80	0.002	0.038

### Moderate exercise protects against osteoarthritis-related pathology at 8 weeks

**FIGURE 3:**
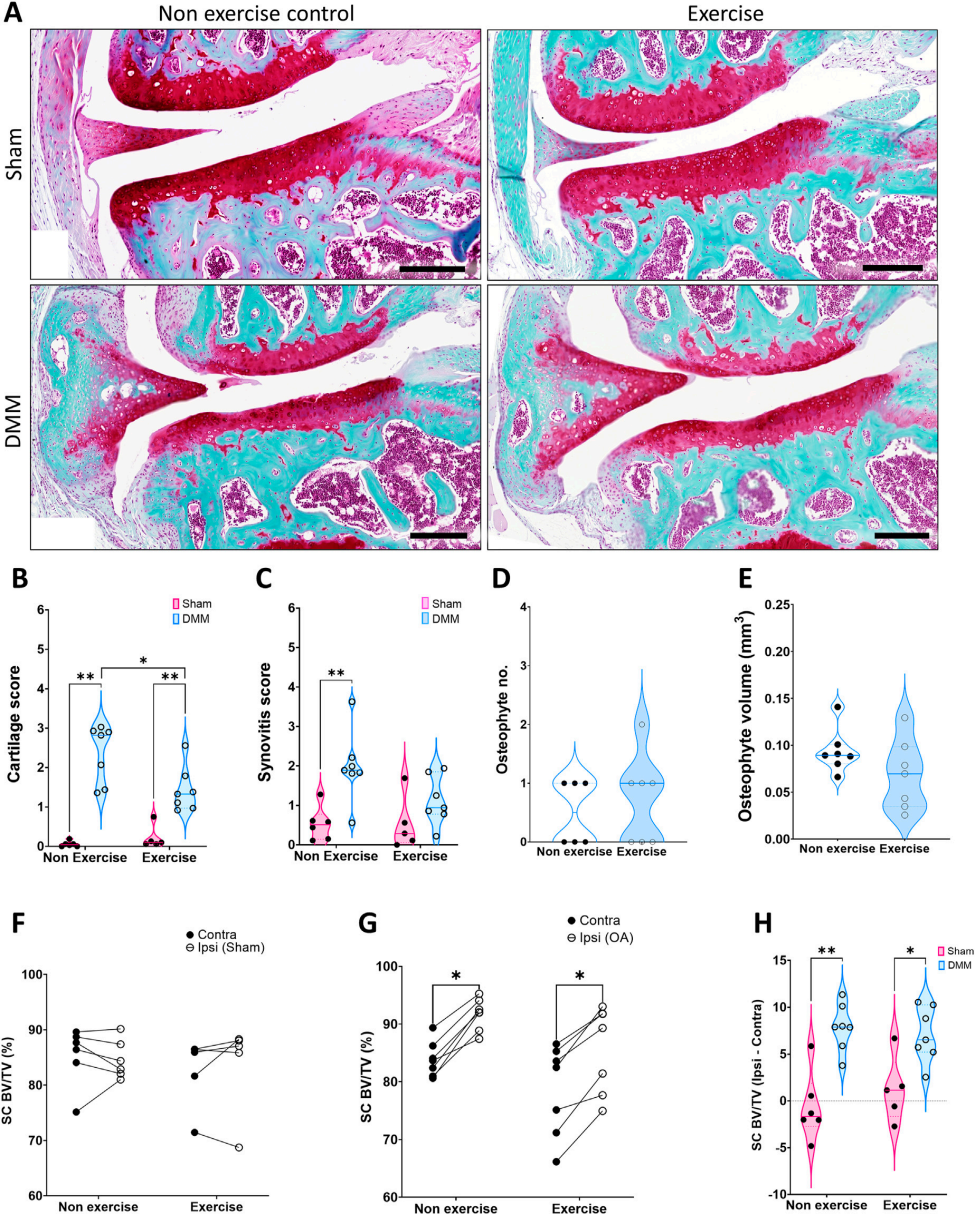
Disease parameters on mice 8 weeks after induction of OA. **(A)** Representative images of the joint at 8 weeks, stained with SafraninO for cartilage and Fast Green for bone. **(B)** Cartilage score. **(C)** Synovitis score. **(D)** Osteophyte number. **(E)** Osteophyte volume. **(F)** Comparison of medial subchondral bone compartment density (% BV/TV) between the operated ipsilateral (Ipsi) and control contralateral (Contra) legs in **(F)** sham and **(G)** DMM groups. **(H)** Change in tibial subchondral bone sclerosis (Ipsi–Contra). Comparison between exercise and non-exercise groups was done with a 2-way ANOVA with Bonferroni correction unless data was not normally distributed, in which case it was then compared by a Mann-Whitney test. Paired comparisons were conducted via a paired t-test. *p < 0.05, **p < 0.01.

While no difference was detected in cartilage damage between exercised and non-exercised groups at 4-week post-surgery, there was lower cartilage damage at 8-week in the exercised group (Figures 3A,B). DMM-driven synovitis was significantly higher than the sham control only in the non-exercise group (Figure 3C), however, this was not significant when comparing to the DMM exercise group (*p* = 0.06). 8 weeks after induction, osteophytes merge with the surrounding bone as the subchondral plate expands, and thus protruding osteophytes are difficult to discern. The outcome of this is that most DMM operated mice presented with one or less visible osteophytes (Figure 3D). Notably, at this time point subchondral bone expansion is clearly visible in 2D images, and quantification revealed that subchondral osteophyte volume was equivalent between the non-exercise and exercise group (Figure 3E).

Medial subchondral bone osteosclerosis was again evident in the DMM ipsilateral leg (Figures 3F,G), yet the change between ipsilateral and contralateral was similar in the non-exercise and exercise group (Figure 3H). However, the increase in trabecular thickness in the subchondral bone was significantly lower in the exercise group (Table 2). Furthermore, tibial trabecular bone of the operated leg was still structurally different only in the exercised DMM group when compared to the contralateral leg 8-week after surgical intervention. The trabecular bone was more connected (Trabecular pattern factor, Tb.Pf.) and more plate-like (structural model index, SMI), yet less organized (degree of anisotropy, DA, Table 2).

## Discussion

In the present study, we used a moderate form of exercise requiring mice to walk 850 m a day, 5 days/week, which had no impact on the normal nocturnal activity. Hence, this did result in a 1.5 fold increase of physical activity in the exercised mice. This protocol allowed for recovery from surgical intervention before the start of forced exercise unlike other reported protocols which were initiated shortly after intervention or later when disease is established. Also, we induced OA by surgical DMM, which is a model of post-traumatic OA. DMM is milder in comparison to other more extreme forms of induction such as ACL-T,less variable than ageing, spontaneous or high fat diet models and resembles a proportion of human OA cases. We assessed whether the selected protocol exerted any physiological benefits. Exercise resulted in a decrease in weight gain and loss of WAT mass, indicating that this form of exercise, although moderate, exerted a physiological effect. This is an important aspect to consider, as it is well established that weight loss reduces risk of OA, as well as improving outcomes in established OA (Messier et al., 2013; Hunter et al., 2015; Panunzi et al., 2021).

Importantly, our induced moderate form of exercise resulted in protection against cartilage damage after 7-week of exercise. In addition to the significant changes in cartilage, evaluation of trabecular bone in the exercise DMM group revealed a more plate-like micro-structure with increased connectivity, similar to findings observed by Hahn et al. (2021), and known for offering higher bone strength (Teo et al., 2007). Moreover, there was an early, albeit temporary, improvement in subchondral bone osteosclerosis in the exercise group; expressed by the significantly smaller increase in bone density of the subchondral bone. It has been shown that increased bone density of the subchondral bone microarchitecture, as induced by PTH dosing, correlated with cartilage degeneration in mice (Orth et al., 2014). We observed a similar correlation where lower cartilage damage corresponded to lower subchondral bone density (Supplementary Figure S2). There was also an initial increase in osteophyte formation, which may indicate an acceleration of the subchondral bone expansion (Iijima et al., 2017) that ensues in the bone adaptation phase of the DMM model to dissipate the increased load. Quantification of the observed end-stage subchondral bone expansion (e.g. osteophyte volume) did not correspond with prior studies (Iijima et al., 2017) where an exercise-induced reduction was shown. This may be due to differences of DMM in rats in comparison to mice. Regardless of this inconsistency, the bone features we show in this study suggest that there is an improvement in the way the damaged joint is loaded in the exercised group. Notably, bone adapts to changes in mechanical loading and the DMM model substantially changes the way the joint is loaded. In essence, instead of the meniscus dissipating the load in the joint, this is transmitted primarily through cartilage and subchondral bone (Das Neves Borges et al., 2014). Thus, the delay in subchondral osteosclerosis we report in the exercise group, together with the change in the microarchitecture of the metaphyseal trabecular bone, suggest that exercise changes the way in which the load is dissipated throughout the joint. In explanation, it is conceivable that load is shifted to the metaphysis rather than subchondral bone. Furthermore, this delay in osteosclerosis might underpin the 8-week cartilage protection we observed. Indeed, it has previously been observed that subchondral bone changes occur rapidly, preceding significant cartilage damage in this OA model (Huesa et al., 2016). In addition to the observed bone changes, prior studies have demonstrated that DMM reduces muscle function four and 8-week post-surgery (van der Poel et al., 2016). It therefore has to be taken into consideration that exercise induced improvement in muscle strength, resulting in joint stabilization and altered load (Knoop et al., 2013; Nha et al., 2013). However, we did not observe any macroscopic changes in muscle mass, thus further studies are required to definitively address this question. Finally, going forward it is also important to consider that the effect of exercise may transcend load and fundamentally influence cellular signaling in the joint environment, which also contributes to the observed pathological changes (Griffin et al., 2012; Hahn et al., 2021; Vadalà et al., 2020).

In summary, this study establishes a model of early moderate exercise that leads to reduced body weight gain, cartilage degradation, delays osteosclerosis, and changes trabecular microarchitecture on a widely used model of OA in mice, thus amenable to mechanistic studies utilizing transgenic animals. Such investigations may be particularly important as exercise programmes may be inappropriate for many patients, and low adherence to long term physiotherapy reduces effectiveness of prescribed exercise (Nicolson et al., 2018). It is important to note that the murine exercise protocol used simulates the situation of a human exercising shortly after sustaining a joint injury of a type likely to induce OA onset. The current study does not, however, address how this type of exercise regime would affect established OA; this is a key question that future studies should address. Furthermore, it will be important to conduct longer-term studies that would indicate if this form of moderate exercise affords long-term or merely transient benefit to the joint tissues.

## Data Availability

The raw data supporting the conclusions of this article will be made available by the authors, without undue reservation.

## Supplementary material

Supplementary Figure S1 Dynamic weight bearing as a measure of surrogate pain (Bioseb, France). Data expressed as the ratio of the load between the ipsilateral and contralateral load (Ipsi/Contra). Data expressed as Mean ± Standard deviation.

Supplementary Figure S2 Correlation of Cartilage damage and subchondral bone density 8 weeks after DMM induction, taking data from both exercise and non-exercise groups.

Supplementary Table S1 Experimental groups in the study.
