# Supplementary material for: Moderate exercise protects against joint disease in a murine model of osteoarthritis
Source: Front Physiol. 2022 Dec 5;13:1065278. doi: 10.3389/fphys.2022.1065278 (PMC9760924; doi:10.3389/fphys.2022.1065278)
Supplement: Supplementary file 1 [file Table1.docx]

Supplemental table 1

|  | Non-exercise | | Exercise | |
| --- | --- | --- | --- | --- |
| Time (weeks) | Sham | DMM | Sham | DMM |
| 4 | 0 | 7 | 0 | 10 |
| 8 | 6 | 7 | 5 | 7 |
