# Supplementary figures and images for: Moderate exercise protects against joint disease in a murine model of osteoarthritis
Source: Front Physiol. 2022 Dec 5;13:1065278. doi: 10.3389/fphys.2022.1065278 (PMC9760924; doi:10.3389/fphys.2022.1065278)

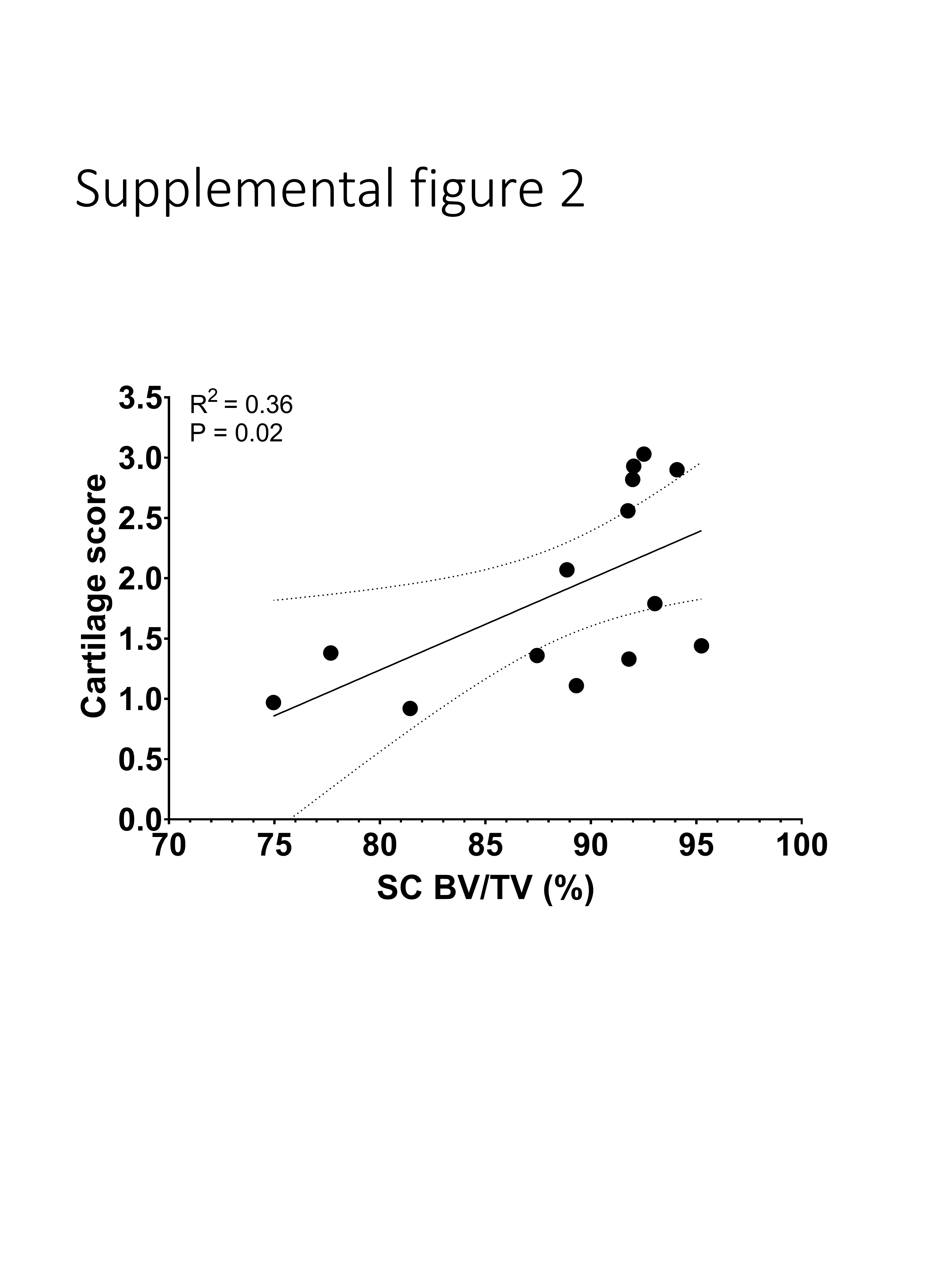

Supplement: Supplementary file 2 [file Image2.TIF]

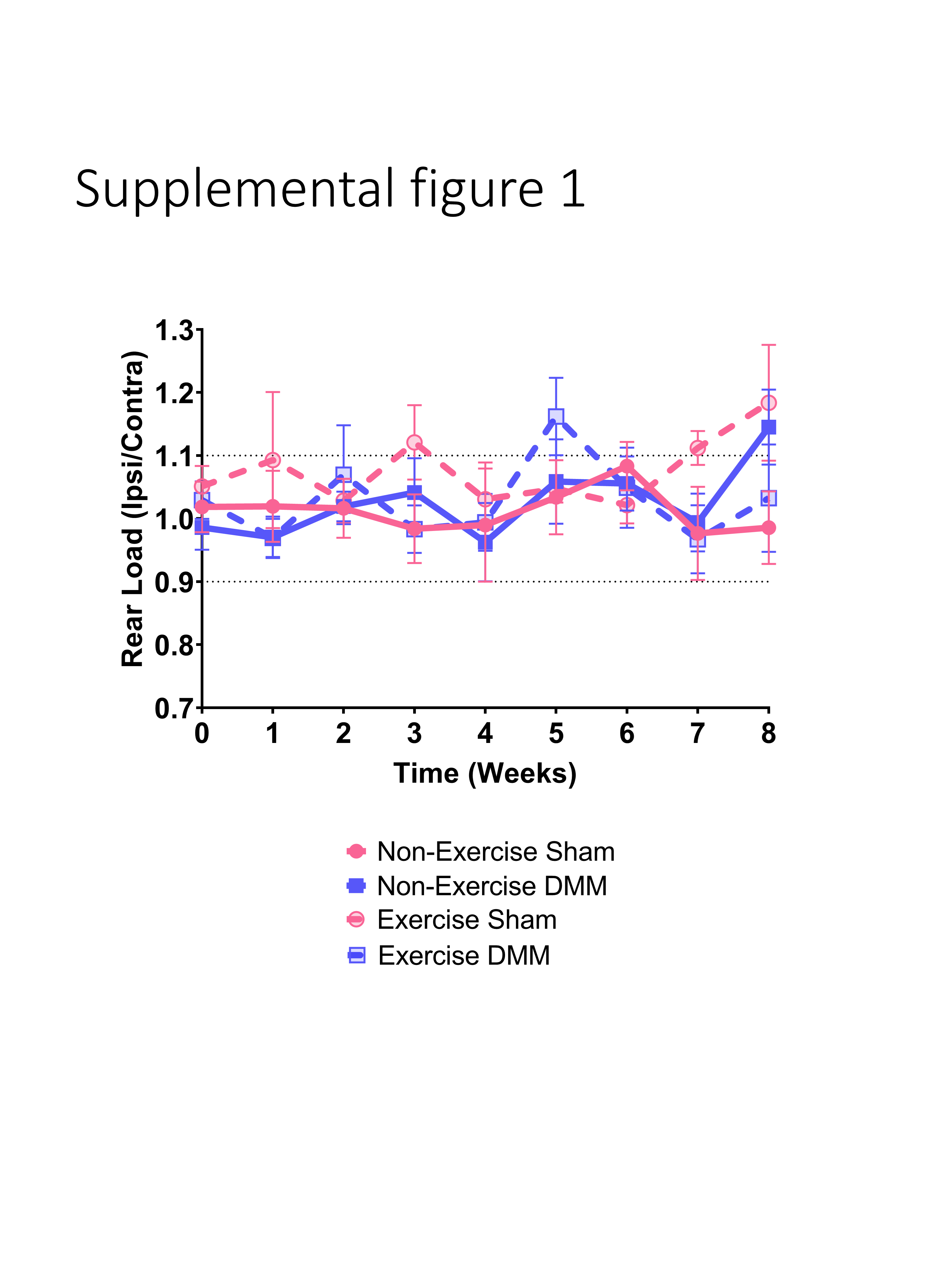

Supplement: Supplementary file 3 [file Image1.TIF]
